# Ethanol-induced conditioned taste aversion and associated neural activation in male rats: Impact of age and adolescent intermittent ethanol exposure

**DOI:** 10.1371/journal.pone.0279507

**Published:** 2022-12-22

**Authors:** Jonathan K. Gore-Langton, Elena I. Varlinskaya, David F. Werner, in part of the Neurobiology of Adolescent Drinking in Adulthood Consortium (NADIA)

**Affiliations:** 1 Center for Development and Behavioral Neuroscience, Binghamton University, Binghamton, New York, United States of America; 2 Department of Psychology, Binghamton University, Binghamton, New York, United States of America; 3 Developmental Exposure Alcohol Research Center, Binghamton, New York, United States of America; Oregon Health and Science University, UNITED STATES

## Abstract

Individuals that initiate alcohol use at younger ages and binge drink during adolescence are more susceptible to developing alcohol use disorder. Adolescents are relatively insensitive to the aversive effects of alcohol and tend to consume significantly more alcohol per occasion than adults, an effect that is conserved in rodent models. Adolescent typical insensitivity to the aversive effects of alcohol may promote greater alcohol intake by attenuating internal cues that curb its consumption. Attenuated sensitivity to the aversive effects of alcohol is also retained into adulthood following protracted abstinence from adolescent intermittent ethanol (AIE) exposure. Despite these effects, much remains unknown regarding the neural contributors. In the present study, we used a conditioned taste aversion (CTA) paradigm to investigate neuronal activation in late-developing forebrain structures of male adolescents and adult cFos-LacZ transgenic rats as well as in AIE adults following consumption of 0.9% sodium chloride previously paired with an intraperitoneal injection of 0, 1.5 or 2.5 g/kg of ethanol. Adults that were non-manipulated or received water exposure during adolescence showed CTA to both ethanol doses, whereas adolescents displayed CTA only to the 2.5 g/kg ethanol dose. Adults who experienced AIE did not show CTA. Adults displayed increased neuronal activation indexed via number of β-galactosidase positive (β-gal+) cells in the prefrontal and insular cortex that was absent in adolescents, whereas adolescents but not adults had a reduced number of β-gal+ cells in the central amygdala. Adults also displayed greater cortical-insular functional connectivity than adolescents as well as insular-amygdalar and prefrontal cortex-accumbens core functional connectivity. Like adolescents, adults previously exposed to AIE displayed reduced prefrontal-insular cortex and prefrontal-accumbal core functional connectivity. Taken together, these results suggest that attenuated sensitivity to the aversive effects of ethanol is related to a loss of an insular-prefrontal cortex-accumbens core circuit.

## Introduction

Alcohol is the most used drug among adolescents worldwide. According to the Monitoring the Future survey, in 2020 more than half (55.3%) of high school seniors had used alcohol in the past year and 16.8% drank at binge levels within the past two weeks [1]. Rates of past-year alcohol use and binge drinking, defined by the National Institute of Alcohol Abuse and Alcoholism as a pattern of drinking that brings blood alcohol concentrations to 0.08 g/dl or above, were lower for 10^th^ graders (40.7 and 9.6%) and 8^th^ graders (20.5 and 4.5%). Several studies have shown that early onset of alcohol use as well as an escalation of drinking during adolescence increases the risk of developing an alcohol use disorder (AUD) in adulthood [2–4]. According to the Substance Abuse and Mental Health Services Administration [5], adolescents and adults display different patterns of alcohol drinking: while adults consume alcohol more frequently than adolescents, adolescents tend to drink substantially more per occasion, with some adolescents demonstrating high-intensity or extreme binge drinking by consuming 10+ and even 15+drinks in a row [6, 7]. (It is unfortunate that alcohol use is relatively high among adolescents, given that this demographic is particularly susceptible to the long-term negative neurocognitive and neurodevelopmental effects of alcohol. For example, binge drinking during adolescence has been associated with impairment of attention, information retrieval, and visuospatial skills [8]. Magnetic resonance imaging (MRI) studies have shown that hippocampal volumes tend to be smaller in adolescents with AUDs than age-matched controls and that this effect is more pronounced with earlier alcohol onset and greater duration of drinking [9, 10].

Given the established harms of adolescent alcohol use, it is important to characterize neural contributors to adolescent-typical binge and high-intensity drinking and to investigate alcohol-induced alterations in the developmental trajectory of the adolescent brain. Therefore, animal models (typically rodents) are a useful tool for understanding adolescent-typical responsiveness to ethanol as well as the neural perturbations caused by adolescent ethanol exposure, which is often not possible with human subjects. Animal models allow for control of several variables, including genetic background, environmental conditions, and ethanol exposure regimens (dose, frequency, age, and duration of exposure), while manipulating only variables of interest (e.g., age, sex, early experience, etc). Animal studies recapitulate that adolescent rats and mice not only consume more ethanol on a g/kg basis than their adult counterparts [11–15] (;, but also are less sensitive than adults to the undesired effects of ethanol that may serve as cues to curb drinking. These adverse effects include ethanol-induced motor impairment [16–18] (, sedation [19, 20], social inhibition [21], and aversion [15, 22–24]. In laboratory rodents, sensitivity to the aversive effects of ethanol has commonly been assessed using a conditioned taste aversion (CTA) paradigm. In this paradigm, ingestion of a novel flavour (conditioned stimulus; CS) is paired with the dysphoric effects of ethanol or other drugs (unconditioned stimulus; US). When an animal is given a subsequent opportunity to consume the CS, it will tend to avoid or reduce its intake, presumably due to the association of the CS with the aversive effects of the drug. The degree to which the animal reduces its consumption relative to a vehicle-injected control is used as an index of the dysphoria experienced during the initial CS-US pairing. Relative to adults, adolescent rats require higher doses [22, 23] (or more CS-US pairings to develop a CTA [22]. As the overall hedonic value of a drug is thought to be a function of the balance between its rewarding and aversive effects [25, 26], the relative insensitivity of adolescents to the aversive effects of ethanol may contribute to high levels of adolescent ethanol intake. Indeed, a meta-analysis of rodent genetic studies confirmed that insensitivity to the aversive effects of ethanol assessed in a CTA paradigm was more strongly related to ethanol consumption than sensitivity to the rewarding effects [27].

Similar insensitivity to the adverse effects of ethanol has been reported in adult laboratory rodents following chronic exposure to ethanol during adolescence. Adult mice and rats exposed to ethanol in adolescence demonstrated attenuated sensitivity to ethanol-induced sedation [28], motor impairment [17], and aversion [29, 30]. It is likely that attenuated sensitivity to some adverse effects of ethanol is rather specific to adolescent ethanol exposure, since attenuation of ethanol sensitivity was not evident following equivalent ethanol exposure and abstinence in adulthood [29, 31]. Furthermore, attenuated sensitivity to ethanol is more pronounced when adolescent ethanol exposure is intermittent rather than continuous, mimicking adolescent-typical binge drinking [32]. Therefore, adolescent intermittent ethanol (AIE) exposure of laboratory rodents is frequently used as an animal model of adolescent binge-like episodes [33, 34]. Route of ethanol administration does not play a substantial role, given that attenuated sensitivity to different ethanol effects in adulthood has been reported following ethanol vapor inhalation [29], intraperitoneal injection [28], and intragastric gavage [30].

Understanding the neural contributors of adolescent typical resistance to the aversive effects of ethanol as well as AIE-associated insensitivity to ethanol-induced CTA may prove important for understanding mechanisms of high-intensity drinking during adolescence and the development of AUDs in later life. To our knowledge, only one study has assessed patterns of neuronal activation using Fos-immunoreactivity in adolescent and adult males following ethanol-induced CTA in response to the US [35]. Although AIE has been shown to attenuate ethanol-induced CTA in male mice [29] and rats [30], no studies to our knowledge have investigated patterns of neuronal activation in CTA-associated brain regions in adults following AIE exposure. Therefore, the present experiments were designed to assess patterns of neuronal activation within brain regions involved in response to the CS following ethanol-induced CTA in adolescent and adult male rats (Experiment 1) as well as in adult males following AIE (Experiment 2).

Male cFos-LacZ transgenic rats on a Sprague-Dawley background were used as experimental subjects. Since these transgenic rats express β-galactosidase (β-gal) under the control of the *cfos* promoter, cFos and β-gal proteins are co-expressed in neurons strongly activated by a certain stimulus and not in the surrounding neurons that are inactive or weakly activated [36]. Therefore, β-gal expression can be used as a proxy for cFos. While cFos expression requires immunohistochemical techniques, β-gal is detected by X-gal staining–a rapid and convenient histochemical assay. In Experiment 1, the regions of interest (ROIs) included the prelimbic cortex (PrL), infralimbic cortex (IL), agranular insula ventral (AIV) and dorsal (AID) subdivisions, nucleus accumbens core (NAcC) and shell (NAcSh), basolateral (BLA) and central (CeA) amygdala. We focused on later developing cortical and subcortical regions (e.g., PFC, NAcC) involved in CTA learning since we hypothesized that these would be more likely to show age-related differences in neuronal activity compared to earlier developing brainstem and more caudal structures [e.g., nucleus tractus solitarius (NTS), parabrachial nucleus (PBN)]. The dorsal striatum (DS) which has no known role in CTA was included as a control ROI. In Experiment 2, neuronal activation by the CS was assessed in the AIV, AID, PrL, IL, NAcC, BLA, and CeA.

## Materials and methods

### Subjects

All experimental subjects were generated from cFos-LacZ transgenic male rats that were backcrossed with outbred Sprague-Dawley females from our animal colony at Binghamton University over multiple generations. Breeders were obtained from the transgenic line of animals originally developed by Dr. Curran while at Roche Institute of Molecular Biology. Experimental animals were from the N7-8 generation (99.9% gene similarity) with our standard Sprague-Dawley breeders or higher. One day after birth [postnatal day (P)1], litters were culled to 8–10 pups, with no more than a 2-pup difference between sexes. On P21, pups were weaned and group-housed with same sex littermates. Between P20 and P24 tissue was collected by ear punch for LacZ genotyping (TransnetYX, Cordova, TN). Only LacZ positive (LacZ+) males were used. Animals were housed in a temperature-controlled (22°C) vivarium on a 12/12 light/dark cycle (lights on at 0700). All animals were treated under guidelines established by the National Institutes of Health and protocols approved by Binghamton University Institutional Animal Care and Use Committee (843–20 and 872–22).

### Conditioned taste aversion (CTA)

The day before conditioning, animals were single-housed and given 50% of the water they consumed the previous day to encourage intake of the CS on conditioning day. On conditioning day, animals were transported in their home cages from the holding room to a testing room containing dim light (15–20 lux) and a white noise generator set at 68 decibels. Home cage tops containing food were replaced with new tops without food. After 15 min of habituation to the room, a single bottle containing 0.9% sodium chloride (NaCl) was placed on the home cage for a 1 h access period. Then bottles were removed and weighed, and animals received an intraperitoneal (i.p.) injection of a single ethanol dose (0, 1.5 or 2.5 g/kg). Animals that drank *less than 0*.*5 g of NaCl* were excluded. Following the ethanol injection, animals were left in the testing room for another 15 min prior to being returned to their holding rooms. At this time, home cage tops were returned, and animals were given full water bottles. The next day animals were given 50% of the water they consumed in their home cage within the past 24 h. The following day CTA testing occurred, with animals again being exposed to the CS (NaCl) for 1 h. After removal of the bottles, rats were left undisturbed for 1 h until they were euthanized for tissue collection.

### Tissue preparation

Animals were euthanized with sodium pentobarbital (Fatal-Plus, Vortech Pharmaceuticals, Dearborn, MI) and perfused with 0.1M phosphate-buffered saline (PBS) at a rate of 30ml/min (200cc for adolescents or 250cc for adults). Then the flow was switched to 4% paraformaldehyde in 0.1M PBS (infused at same volume as PBS) to fix the tissue. Immediately after fixation, the brain was extracted and placed into a vial containing 4% paraformaldehyde solution for a 90 min post-fix period. The brain was then transferred into a cryoprotective solution of 30% sucrose in water until saturated and temporarily stored at -20°C until the brain was sectioned and stained.

All collected brains prior to being coronally sectioned were flash-frozen in methyl-butane at -80°C. Once brains were prepared to be sectioned the cerebellum was removed with a razor blade and the brain was mounted to a chuck by applying OCT gel and frozen between temperatures of -20 to -16°C prior to slicing. Slices were collected every 30μm and transferred into 1.7ml microcentrifuge tubes containing antifreeze solution (30% ethylene glycol and 30% glycerol in 0.5M PBS), then stored at -20°C until X-Gal histochemistry was performed. A serial collection method was used in which every 12^th^ slice (360μm between each collection) was placed into the same tube for a total of 6 slices per tube.

### X-gal histochemistry

On the first day of the X-Gal enzymatic reaction procedure, representative brain regions were gently agitated in X-Gal fix buffer (2mM magnesium chloride and 5mM EGTA in 0.1M PBS) followed by two washes in X-Gal wash buffer (2mM magnesium chloride in 0.1M PBS). Next, slices were placed into X-Gal staining solution (2mM magnesium chloride, 5mM potassium ferrocyanide, 5mM potassium ferricyanide, and 1 mg/ml of X-Gal in 0.1M PBS) and left to incubate overnight in a 37°C orbital stage chamber. On the second day, slices were again washed twice in X-Gal wash buffer and mounted onto chromalum-gelatin-coated slides and left to air-dry over night at room temperature. The next day, mounted slices were counterstained in eosin and dehydrated using a series of ascending percentages of ethanol, then cleared with Xylene and coverslipped.

### Regions of interest (ROI) and cell counting

In Experiment 1, imaging was conducted using a Keyence BZ-X800 microscope (Keyence corporation, Itasca, IL) and stained soma were quantified using NIH Image J software. ROIs included the PrL, IL, AIV, AID, NAcC, NAcSh, BLA, CeA, and DS. β-gal+ cells were manually counted by the same experimenter within a set size grid box for each ROI (Table 1). Several studies which have used a CTA paradigm have demonstrated synchronized cFos expression or anatomical connections between cortico-amygdalar structures [37–41]. The rat brain atlas [42] was used as a guide for grid placement and size. All cells found on the border of the grid box (i.e., partially obscured by border) were counted. Across experiments 1 and 2 there was an average of 3–5 section/region/animal, with a range of 1–13 slices. A unilateral hemisphere of a coronal slice was considered as a single section. In Experiment 2, ROIs included the PrL, IL, AIV, AID, NAcC, BLA, and CeA. The NAcSh was excluded due to recent reported effects along the rostro-caudal axis [43]. Imaging was done using an Olympus VS200 slide scanner (Olympus corporation, Tokyo, Japan) and stained soma were quantified using HALO 3.0 cytonuclear module (Indica Labs, Albuquerque, NM). Parameters for the HALO analysis algorithm for detection of a β-gal+ expressing cell were set at a minimum optical tissue density of 0.037 and minimum nuclear area of 10. Comparisons between cell counts between manual counting methods versus Halo did not reveal any significant differences in any of the assessed regions. All β-gal+ cell counts were standardized to an area of 1mm^2^ to make graphical comparisons between cell density in different ROIs.

**Table 1:** Brain region coordinates based on the Paxinos and Watson Atlas (2007).

Region of Interest (ROI)	Grid Size	Coordinates from Bregma
AIV	0.2 × 1 mm (0.2 mm^2^)	4.20 to 2.28 mm
AID	0.2 × 1 mm (0.2 mm^2^)	4.20 to 2.28 mm
PrL	0.5 mm^2^	5.16 to 2.76 mm
IL	0.25 mm^2^	3.72 to 2.76 mm
NAcC	0.4 mm^2^	2.76 to 0.72 mm
NAcSh	0.4 mm^2^	2.52 to 0.72 mm
BLA	0.5 mm^2^	‒1.72 to -3.24 mm
CeA	0.5 mm^2^	‒1.72 to 3.00 mm
DS	0.5 mm^2^	2.52 to 0.00 mm

### Experiment 1: Design and statistical analyses

**Fig 1:**
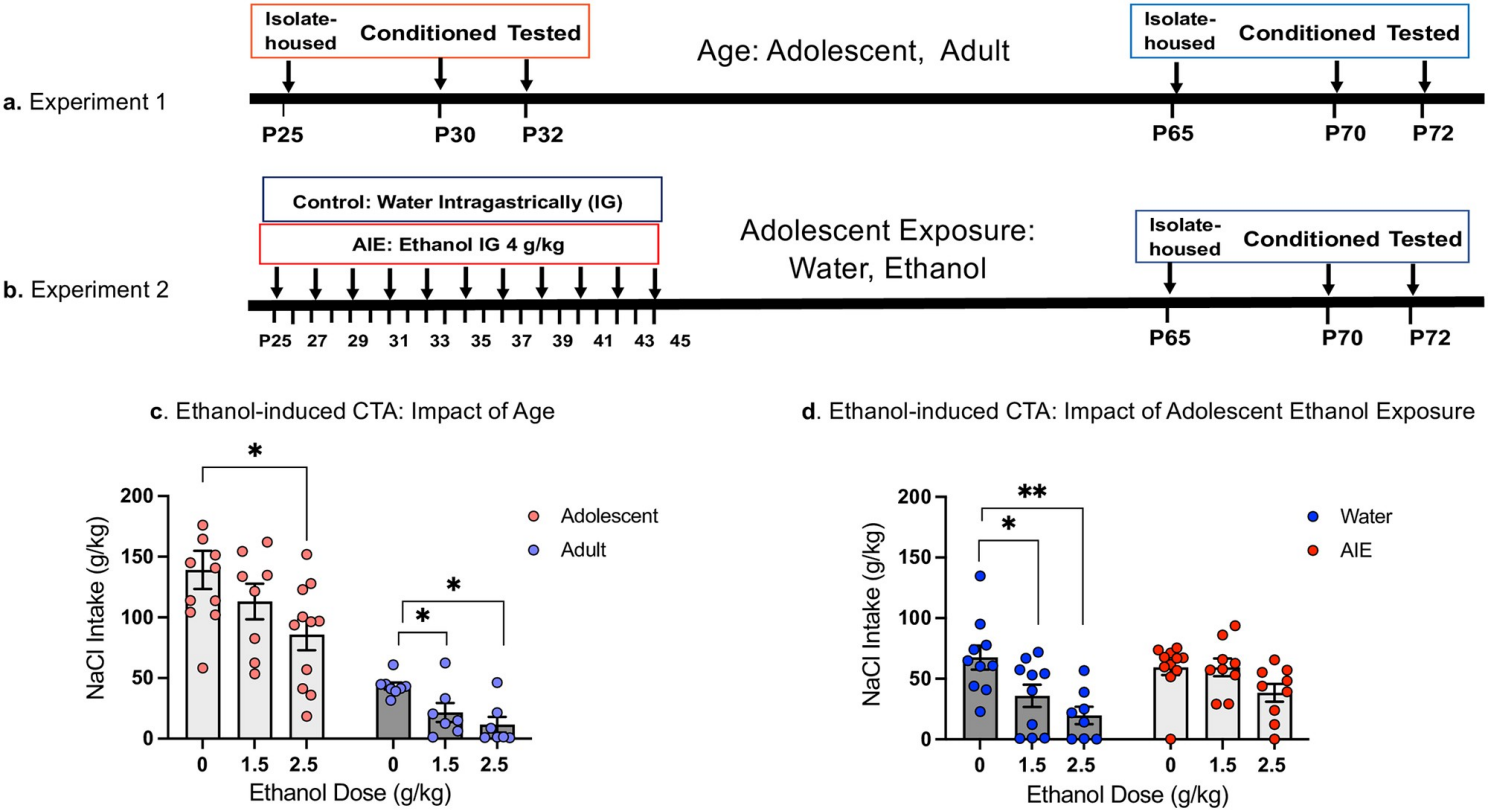
Timelines and NaCl (CS) intake on test day in Experiment 1 and Experiment 2. Timelines for Experiments 1 and 2, respectively (a, b), and NaCl (CS) intake (g/kg) on test day (c, d, respectively) are shown. Asterisks depict significant changes in NaCl intake on test day within each Age relative to controls conditioned with saline (0 g/kg EtOH dose), *—p < 0.05, **—p <0.01.

Experiment 1 was designed to assess the impact of age on neuronal activation in CTA-associated brain regions following re-exposure to the CS. The design was a 2 Age (adolescent, adult) X 3 Ethanol Dose (0,1.5, 2.5) factorial. Previous studies from our research group have demonstrated age-dependent differences in CTA sensitivity with adults developing CTA at both the 1.5 and 2.5 g/kg ethanol doses, whereas adolescents only develop CTA at the 2.5g/kg ethanol dose [23, 35]. Animals were single-housed on P25 for animals tested in adolescence and P65 for animals tested in adulthood. Conditioning occurred on P30 (adolescents) or P70 (adults), with CTA testing and brain tissue collection occurring two days later on P32 or P72 (see Fig 1a).

Age-related differences in body weights between adolescents and adults were compared using an independent samples t-test. Intake of the CS (g/kg) on both conditioning and test day were assessed using 2 Age (i.e., adolescent, adult) X 3 Ethanol Dose (0,1.5, 2.5 g/kg) ANOVAs. When main effects of Age or Ethanol Dose were evident, separate one-way ANOVAs for each age were conducted to assess whether these main effects were driven by one age only. This approach allowed us to determine ethanol doses effective in eliciting CTA within each age prior to assessing neuronal activation. Ethanol-induced CTA was determined as a significant decrease in intake from the age-appropriate saline (0 g/kg) control group [35] using Fisher’s pair-wised post-hoc tests. β-gal expression was examined in each ROI using a 2 Age X 3 Ethanol Dose factorial ANOVA. In the case of age differences indexed via a main effect of Age, similar to CTA behaviour, omnibus ANOVAs were followed by separate one-way ANOVAs for each age, supplemented with Fisher’s LSD tests. A forward multiple stepwise regression was conducted separately for each age allowing for investigation of the influence of each brain region (i.e., β-gal expression) on the strength of CTA (i.e., test day intake). Each ROI was added into the analysis as independent variables in a stepwise manner, with nonsignificant variables being removed at subsequent steps. The F to enter value was 1 and the F to remove value was 0. Patterns of neuronal activation across brain regions were investigated using Pearson bivariate correlations between β-gal in each region within each Age. A false discovery rate of 5% (q-value threshold = 0.05) was maintained using the two-stage set up method of Benjamini, Krieger and Yekutieli to limit the number of false positive comparisons. One outlier animal (adolescent 0 g/kg that was +2 SDs greater than the group mean in several distinct ROIs (AID, AIV, IL, NAcSh, BLA) was excluded from the correlation matrix analysis.

All analyses were separated by Age as factorial ANOVAs revealed greater β-gal expression in adolescents compared to adults in all ROIs except for the BLA where there was a trend for greater expression in adolescents (*p* = 0.08).

### Experiment 2: Design and statistical analyses

Experiment 2 was designed to assess the effect of adolescent intermittent ethanol (AIE) exposure on neuronal activation in CTA-associated brain regions following re-exposure to the CS. The design was a 2 Adolescent Exposure (water, AIE) X 3 Ethanol Dose (0,1.5, 2.5 g/kg) factorial. A study from our research group has shown that a 2.5 g/kg, but not a 1.5 g/kg ethanol dose induces CTA in AIE exposed animals > 3 weeks post AIE [30]. Experimental subjects were given either tap water or 4 g/kg ethanol (AIE) intragastrically (25% v/v in tap water) every other day between P25 and P45 for a total of 11 exposures. All animals that were housed in the same cage received the same exposure (water or AIE). After the last exposure on P45, experimental subjects were left undisturbed for 20 days. Animals remained group-housed with no more than 4 animals per cage until P65 at which point they were single-housed. Animals were conditioned on P70 and tested for CTA on P72 (see Fig 1b).

Body weights of Water and AIE exposure groups were compared across Days (P25-45) using a repeated measures ANOVA during the AIE exposure period, or with independent samples t-test during behavioural testing in adulthood. Intake of the CS on both conditioning and test day were assessed by separate 2 Adolescent Exposure (Water, AIE) X 3 Ethanol Dose (0,1.5, 2.5 g/kg) ANOVAs. Fisher’s LSD tests were used to verify effective doses of ethanol within each adolescent condition for producing CTA as has been observed previously [30]. Similar to Experiment 1, given that our focus was on *a priori* dose-responses to verify onset of CTA within each adolescent condition prior to assessing neural activity, omnibus ANOVAs were followed by separate one-way ANOVAs that allowed us to assess the alterations in sensitivity to the aversive effects of ethanol resulting from AIE. Number of β-gal+ cells was examined in each ROI using a 2 Adolescent Exposure (Water, AIE) X 3 Ethanol Dose (0,1.5, 2.5 g/kg) ANOVA. Omnibus ANOVAs were followed by separate one-way ANOVAs for each Exposure in the case of main effects of Exposure and/or ethanol Dose. In the case of main effects of Adolescent Exposure and/or Ethanol Dose, separate one-way ANOVAs for each Exposure were used followed by Fisher’s LSD *post-hoc* tests. A forward multiple stepwise regression was conducted separately for each Adolescent Exposure condition in order to assess the influence of each brain region (i.e., β-gal expression) on the strength of CTA (i.e., test day intake). Each ROI was added into the analysis as independent variables in a stepwise manner, with nonsignificant variables being removed at subsequent steps. The F to enter value was 1 and the F to remove value was 0. All results were considered statistically significant at *p*≤0.05. Finally, potential functional connectivity between brain regions was investigated using Pearson bivariate correlations between β-gal in each region for each Exposure. A false discovery rate of 5% (q-value threshold = 0.05) was maintained using the two-stage set up method of Benjamini, Krieger and Yekutieli to limit the number of false positive comparisons.

## Results

### Experiment 1. CTA and neuronal activation in associated brain regions following re-exposure to CS: Impact of age

#### Body weights at conditioning day and test day

On conditioning day, the mean body weight for adults (389.55 g ± 7.23 g) was significantly greater than the mean body weight for adolescents (117.30 g ± 1.93 g) (t_50_ = -41.5, p < .0001). On test day, the mean body weight for adults (384.25 g ± 7.62 g) was significantly greater than the mean body weight for adolescents (128.71 g ± 2.22 g) (t_50_ = -36.49, p < .0001).

#### Ethanol-induced CTA

The intake of sodium chloride (CS) on conditioning day did not differ as a function of Age and Ethanol Dose (all p >0.05), with adolescents and adults ingesting comparable amounts of the CS (14.4 ± 0.7 g and 16.1± 1.6 g, respectively). Given baseline differences in body weights, initial between age comparisons were made by calculating intake on a g/kg basis. On test day, the 2 Age X 3 Ethanol Dose ANOVA of the CS intake revealed main effects of Age (*F*_1,46_ = 70.66, *p*< 0.0001) and Ethanol Dose (*F*_2,46_ = 6.03, *p*<0.005), with adolescents consuming significantly more of the CS on test day (112.59 g/kg ± 9.16 g/kg) than adults (26.29 g/kg ± 4.36 g/kg) when data was collapsed across Ethanol Dose (*t*_50_ = 7.60, *p* < 0.0001). Follow-up separate for each age one-way ANOVAs revealed a main effect of Ethanol Dose for both adolescents (*F*_2,27_ = 3.70, *p*<0.05) and adults (*F*_2,19_ = 7.95, *p*<0.01). Post-hoc tests showed that adults developed CTA to both the 1.5 and 2.5 g/kg ethanol doses, demonstrating significantly lower intake of NaCl than their saline control group, while in adolescents, CTA was evident only following the 2.5 g/kg dose (Fig 1c). These results indicate that adolescent cFos-LacZ transgenic male rats were less sensitive to ethanol-induced CTA than their adult counterparts.

#### Neuronal activation

**Fig 2:**
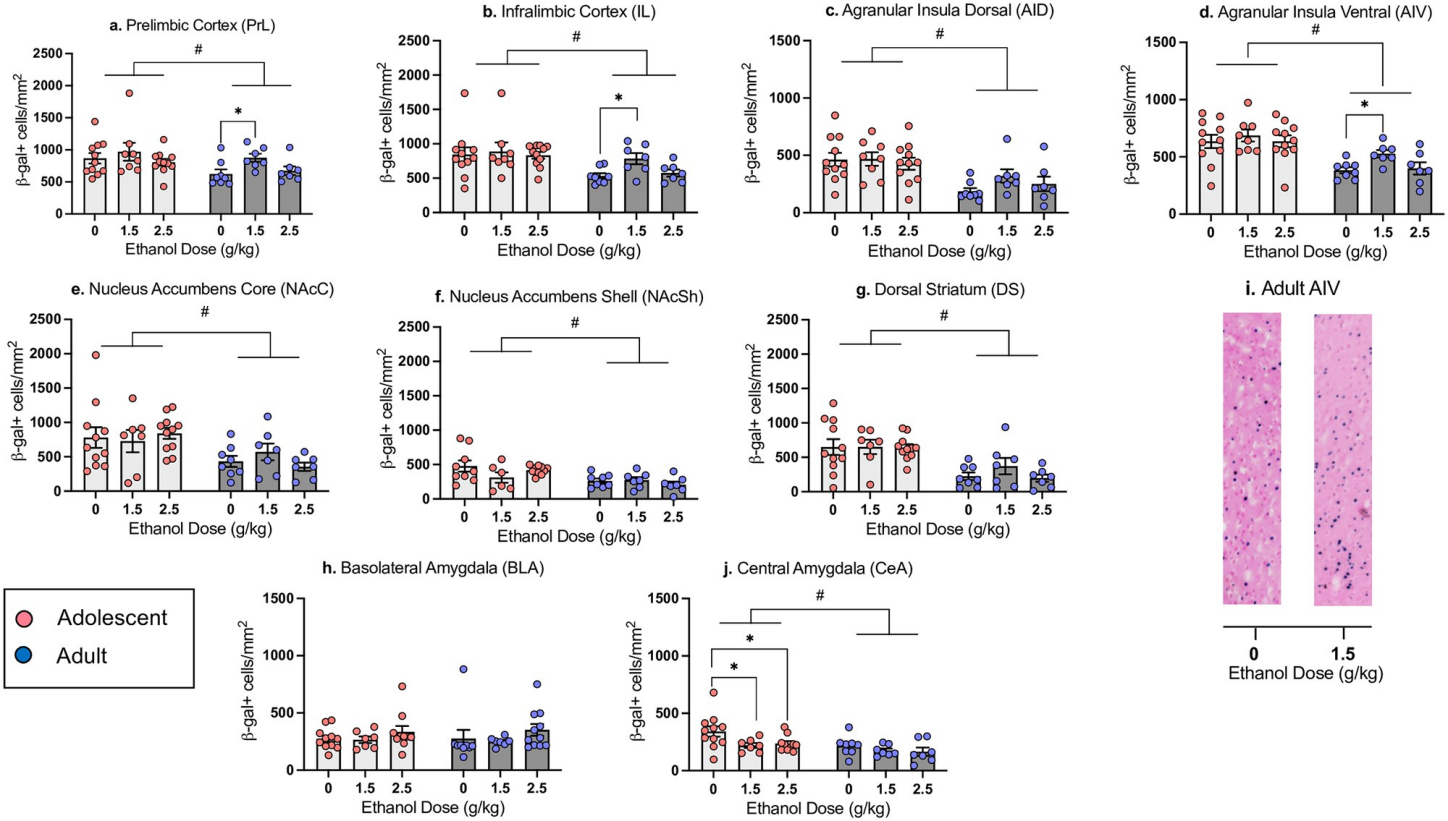
Neuronal activation indexed via number of β-gal+ cells in adolescents and adults. β-gal+ cell counts in the Prelimbic Cortex (a), Infralimbic Cortex (b), Dorsal Agranular Insular Cortex (c), Ventral Agranular Insular Cortex (d), Nucleus Accumbens Core (e), Nucleus Accumbens Shell (f), Dorsal Striatum (g), Basolateral Amygdala (h), and Central Amygdala (j) in adolescent and adult males previously conditioned with 0, 1.5 or 2.5 g/kg ethanol on test day following exposure to the CS (NaCl). (i) Images of β-gal+ cells in AIV of adult rats, 1mm in length. n = 7-11/group; # denotes main effect of age, asterisks (*) mark significant ethanol-associated changes relative to controls conditioned with with saline (0 g/kg ethanol dose) within each Age (p < 0.05).

*Prelimbic cortex (PrL) and infralimbic cortex (IL)*. The ANOVA of β-gal expression in the PrL revealed a main effect of Age (*F*_1,46_ = 5.09, *p*<0.01), with significantly more β-gal+ cells evident in the PrL of adolescents than adults (218.04 ± 12.75 and 180.31 ± 10.87, respectively, *t*_50_ = 2.15, *p* < 0.05, see Fig 2a). Follow-up one-way ANOVAs revealed a main effect of Ethanol Dose in adults (*F*_2,19_ = 3.96, *p*<0.05), but not adolescents. Adults that received the 1.5 g/kg ethanol dose on conditioning day had significantly more β-gal+ cells in the PrL than saline controls (see Fig 2a).

In the IL, the omnibus ANOVA revealed a main effect of Age (*F*_1,46_ = 9.95, *p*<0.01), with a greater number of β-gal+ cells in adolescents than adults (52.16 ± 3.05 and 39.14 ± 2.50, respectively, *t*_50_ = 3.14, *p* < 0.01, Fig 2b). One-way ANOVAs revealed a main effect of Ethanol Dose in adults (*F*_2,19_ = 5.29, *p*<0.050, but not adolescents, with adults in the 1.5 g/kg dose group demonstrating significantly more β-gal+ cells relative to their saline control group, with no changes evident in the 2.5 g/kg ethanol dose condition (see Fig 2b).

*Dorsal (AID) and ventral (AIV) agranular insular cortex*. In the AID, the two-factor ANOVA revealed a main effect of Age (*F*_1,46_ = 18.62, *p*<0.01), with a significantly higher number of β-gal+ cells in adolescents than adults (90.22 ± 6.42 and 49.84 ± 6.07, respectively, *t*_50_ = 4.42, *p* < 0.001, Fig 2c). One-way ANOVAs did not reveal an effect of Ethanol Dose in either adolescents or adults. Similarly, the factorial ANOVA of β-gal expression in the AIV revealed a significant main effect of Age (*F*_1,46_ = 26.13, *p*<0.01). The number of β-gal+ cells in the adolescent AIV was significantly higher than in the adult AIV (129.87 ± 6.22 and 86.91 ± 5.04, respectively, *t*_50_ = 5.08, *p* < 0.001, Fig 2d). One-way ANOVAs within each Age revealed a main effect of Dose only in adults (*F*_2,19_ = 4.21, *p*<0.05), with adults conditioned with the 1.5 g/kg ethanol dose demonstrating significantly more β-gal+ cells than saline controls (see Fig 2i).

*Nucleus accumbens core (NAcC)*, *nucleus accumbens shell (NAcSh)*, *and dorsal striatum (DS)*. In the NAcC, the factorial ANOVA revealed a main effect of Age (*F*_1,45_ = 11.02, *p*<0.01), with β-gal+ cell counts in adolescents being significantly higher than in adults (126.57 ± 11.70 and 72.45 ± 8.52, respectively, *t*_49_ = 3.52, *p* < 0.001, Fig 2e). One-way ANOVAs did not reveal effects of Ethanol Dose at either age. In the NAcSh, there was a main effect of Age (*F*_1,42_ = 13.04, *p*<0.01) as well, with adolescents demonstrating significantly higher β-gal+ cell counts than adults (68.35 ± 5.60 and 39.76 ± 3.62, respectively, *t*_46_ = 3.92, *p* < 0.001, Fig 2f). Follow-up ANOVAs did not reveal effects of Ethanol Dose in either adolescents or adults. In the DS, β-gal+ cell counts only differed as a function of Age (*F*_1,45_ = 25.93, *p*<0.01), with adolescents demonstrating higher β-gal expression than adults (161.40 ± 13.05 and 65.79 ± 11.63, respectively, *t*_49_ = 5.28, *p* < 0.001, Fig 2g). One-way ANOVAs did not reveal effects of Ethanol Dose in either adolescents or adults.

*Basolateral amygdala (BLA) and central amygdala (CeA)*. In the BLA, β-gal+ cell counts did not differ as a function of Adolescent Exposure or Ethanol Dose (see Fig 2h). In the CeA, the ANOVA of β-gal expression revealed a main effect of Age (*F*_1,44_ = 8.96, *p*<0.01) and Ethanol Dose (*F*_2,44_ = 3.75, *p*<0.05). One-way ANOVAs showed a main effect of Dose (*F*_2,25_ = 3.45, *p*<0.05) in adolescents, but not in adults, with adolescents previously conditioned with either the 1.5 or 2.5 g/kg ethanol dose showing significant decreases in β-gal+ cells relative to controls conditioned with saline (Fig 2j).

*Forward-stepwise multiple regression*: *β-gal and test day intake*. In adolescents the forward stepwise multiple regression model revealed a significant positive relationship between β-gal expression in the AID and test day intake (adjusted R^2^ = 0.136, *p* = 0.028). In other words, greater β-gal expression in the AID of adolescents was related to greater intake of the CS on test day. There were no other significant predictors of test intake in the adolescent model. In adults, there was a significant negative relationship between β-gal expression in the BLA and test day intake (adjusted R^2^ = .134, *p* = 0.025). All other ROIs did not differ from the model by the stepwise procedure.

*Patterns of neuronal activation*: *Correlation matrix*. Pearson bivariate correlations were conducted using pairwise comparisons of β-gal+ cell counts in each ROI (see Fig 4a). In both adolescents and adults, significant positive correlations (all *p* < 0.05) were evident between the PrL and IL, PrL and AIV, IL and AIV, AID and AIV, as well as NAcC and NAcSh. Adults displayed increased functional connectivity over adolescents, with significant positive correlations evident between the IL and AID, IL and NAcC, IL and DS, AID and BLA, AIV and BLA, NAcC and DS, NAcSh and DS.

### Experiment 2. CTA and neuronal activation in associated brain regions following re-exposure to the CS: Impact of prior history of adolescent intermittent ethanol (AIE) exposure

#### Body weights at conditioning and test day

Analysis of body weight change during the adolescent exposure period (i.e., P25-P45) with a between-subjects factor of Adolescent Exposure (Ethanol vs. Water) and within-subjects factor of Day revealed main effects of Adolescent Exposure (F_1,55_ = 4.81, p<0.05) and Day (F_10,550_) = 5137.52, p<0.000), but no interaction of Adolescent Exposure X Day. When collapsed across day, throughout the adolescent period AIE animals weighed slightly less than water-exposed controls collapsed across Day (166.76 g ± 1.89 g vs.173.07 g ± 2.52 g, respectively). However, this slight difference in body weight was abolished once subjects were adults, as no differences in body weight between AIE or water-exposed animals were noted on conditioning day (AIE 388.75 g ± 5.34 g vs. water 378.37 g ± 4.85 g) or test day (AIE 386.87 g ± 5.49 g vs. water 373.59 g ± 4.42 g).

#### Ethanol-induced CTA

The intake of sodium chloride (CS) on conditioning day differed as a function of Adolescent Exposure (F_1,51_ = 6.18, *p* < 0.05), with AIE-exposed males consuming significantly (*t*_55_ = 2.41, *p* < 0.05) more than their water-exposed counterparts (25.43 g ± 1.35 g vs. 20.39 g ± 1.60 g, respectively). On test day, the 2 Adolescent Exposure X 3 Ethanol Dose ANOVA of CS intake revealed a significant main effect of Ethanol Dose (*F*_2,51_ = 8.99, *p*<0.01), with rats that were conditioned either with the 1.5 or 2.5 g/kg dose demonstrating significantly lower intake of the CS at test relative to those conditioned with the 0 g/kg ethanol dose, as well as a suggestive effect of Adolescent Exposure (*F*_1,51_ = 4.60, *p* = 0.09). Follow-up one-way ANOVAs conducted within each Adolescent Exposure revealed a main effect of Ethanol Dose only for water-exposed animals (*F*_2,25_ = 7.018, *p*<0.01) animals, with no effect of Ethanol Dose evident in AIE-exposed rats (*F*_2,26_ = 2.951, *p* = 0.07), suggesting that AIE exposure attenuated sensitivity to ethanol-induced CTA in adulthood. P*ost-hoc* tests showed that water-exposed controls demonstrated CTA at both the 1.5 (p < 0.05) and 2.5 g/kg (p < 0.01) ethanol dose (see Fig 1d).

#### Neuronal activation

**Fig 3:**
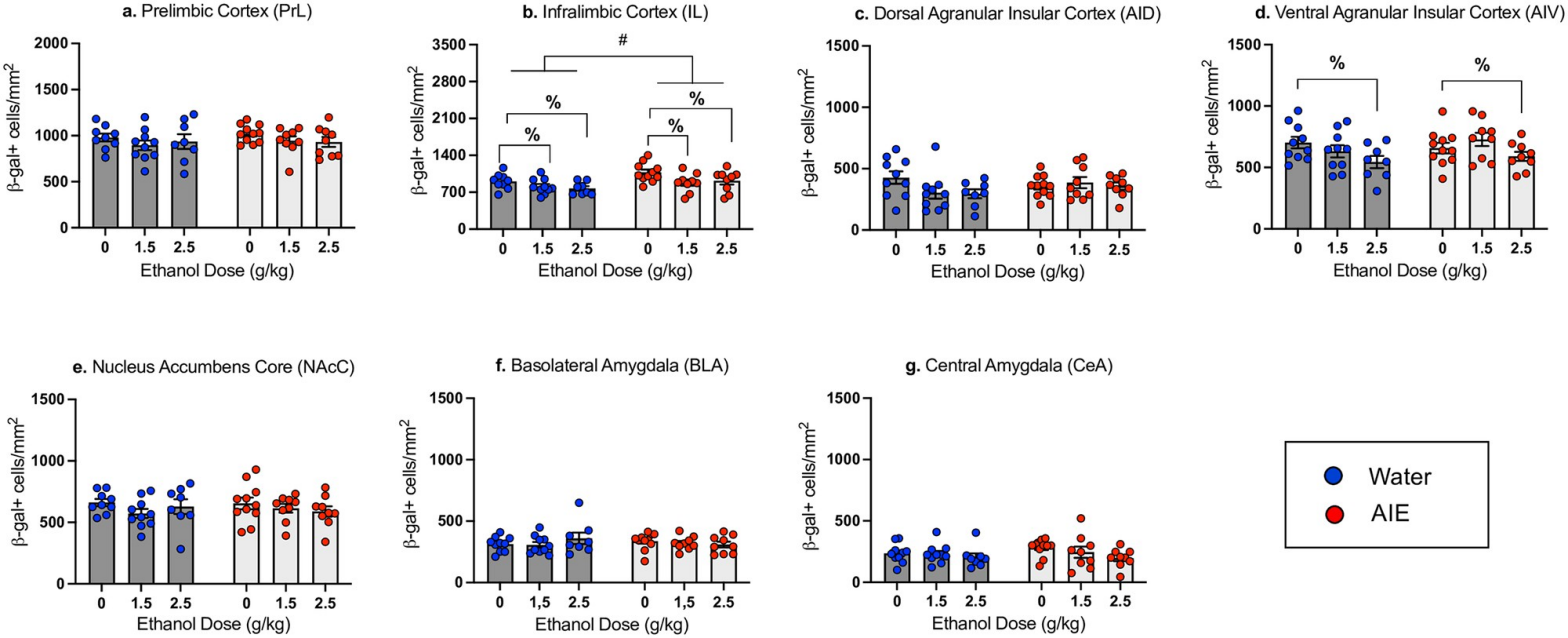
Neuronal activation indexed via number of β-gal+ cells in adults exposed to water or AIE. β-gal+ cell counts in the Prelimbic Cortex (a), Infralimbic Cortex (b), Dorsal Agranular Insular Cortex (c), Ventral Agranular Insular Cortex (d), Nucleus Accumbens Core (e), Basolateral Amygdala (f), and Central Amygdala (g) in adult males exposed to water or ethanol (AIE) during adolescence and previously conditioned with 0, 1.5 or 2.5 g/kg ethanol on test day following exposure to the CS (NaCl). n = 8-10/group; average number of slices per region for individual animal = 3–7. #—denotes main effect of Adolescent Exposure, %marks significant ethanol-associated changes relative to controls conditioned with with saline (0 g/kg ethanol dose) when collapsed across Adolescent Exposure (p < 0.05).

*Prelimbic cortex (PrL) and infralimbic cortex (IL)*. The number of β-gal+ cells in the PrL did not differ as a function of Adolescent Exposure and Ethanol dose (see Fig 3a). However, in the IL a two-way ANOVA revealed a main effect of Adolescent Exposure (*F*_1,50_ = 8.21, *p* < 0.01), with AIE-exposed animals demonstrating more β-gal+ cells than their water-exposed counterparts (60.22 ± 2.31 and 51.89 ± 1.73, respectively), and a main effect of Ethanol Dose (F_2,50_ = 4.99, *p* <0.05), with significant decreases in β-gal expression evident in animals conditioned with either the 1.5 or 2.5 g/kg ethanol dose when collapsed across Adolescent Exposure. Follow-up one-way ANOVAs within each Exposure group did not reveal effects of Dose in the water exposed group and only a suggestive effect in the AIE exposed group (*p* = 0.057) (See Fig 3b).

*Dorsal (AID) and ventral (AIV) agranular insular cortex*. The factorial ANOVA for the AID β-gal expression did not reveal any main effects or an interaction (see Fig 3c). In contrast, β-gal expression in the AIV differed as a function of Ethanol Dose (F_2,51_ = 3.598, *p*<0.05), with a significant decrease in β-gal expression relative to saline controls evident in animals conditioned with 2.5 g/kg ethanol, when collapsed across Adolescent Exposure condition (see Fig 3d). However, one-way follow-up ANOVAs conducted separately for each Adolescent Exposure did not reveal main effects of Ethanol Dose.

*Nucleus accumbens core (NAcC)*, *basolateral amygdala (BLA) and central amygdala (CeA)*. The factorial ANOVAs of β-gal expression in the NAcC, BLA, and CeA did not reveal main effects of Exposure, Dose, or interactions of Exposure X Dose (see Fig 3e–3g).

*Forward-stepwise multiple regression*: *β-gal expression and test day intake*. In AIE exposed animals, the forward stepwise multiple regression model revealed a significant positive relationship between β-gal expression in the AID and test day intake (adjusted R^2^ = 0.435, *p* = 0.007), with greater β-gal expression in the AID related to greater intake of the CS on test day. There were no other significant predictors of test intake in the AIE group. In water exposed subjects, there were significant positive relationships between test day intake and β-gal expression in the AIV (adjusted R^2^ = 0.639, *p* = 0.022) and AID (adjusted R^2^ = 0.639, *p* = 0.039). All other ROIs did not differ from the model by the stepwise procedure.

**Fig 4:**
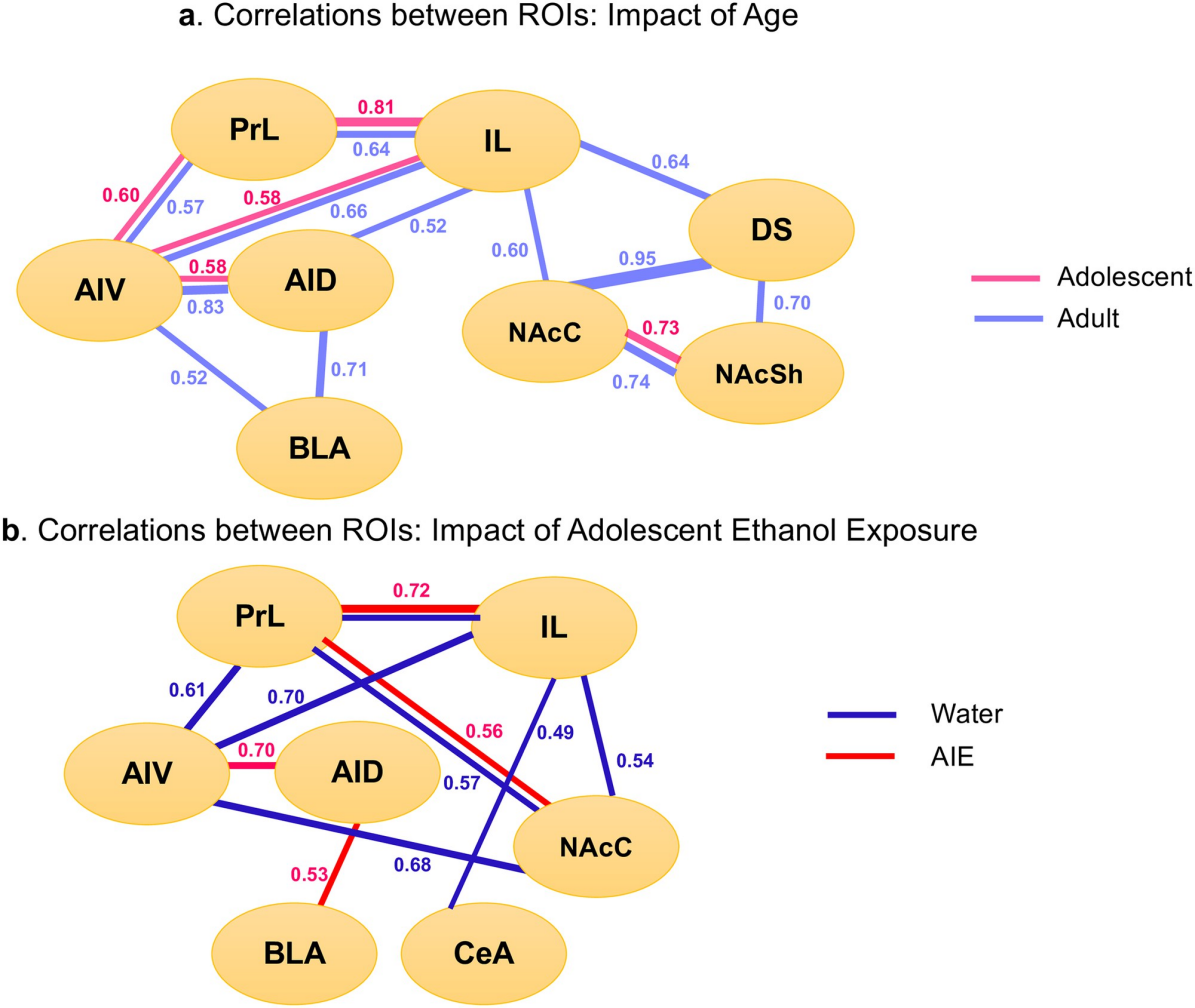
Correlation matrices as a measure of functional connectivity between brain regions. Functional connectivity is shown for adolescents and adults in Experiment 1 (a) and water and AIE exposed adults in Experiment 2 (b). Only significant (p <0.05) correlations are presented, number represent r values.

*Patterns of neuronal activation*: *Correlation matrix*. As in Experiment 1, Pearson bivariate correlations were conducted using pairwise comparisons of β-gal+ cell counts in each ROI (see Fig 4b). In water and AIE exposed animals, significant positive correlations (all *p* < 0.05) were evident between the PrL and IL as well as the PrL and NAcC. In the water-exposed condition, significant positive correlations were also evident between the PrL and AIV, IL and AIV, AIV and NAcC, IL and NAcC, as well as the IL and CeA. In AIE animals, significant positive correlations were evident between the AID and AIV as well as the AID and BLA.

## Discussion

The results of the present experiments demonstrate attenuated sensitivity to ethanol-induced CTA in adolescent males relative to their adult counterparts as well as adult male rats following AIE exposure in early adolescence relative to water exposed control males. While adults in Experiment 1 and water-exposed controls in Experiment 2 demonstrated CTA following conditioning with both doses of ethanol, adolescents developed CTA only following the highest dose, whereas AIE-exposed adults were insensitive to ethanol-induced CTA. The observed age-related difference is in agreement with previous studies that have shown attenuated sensitivity to ethanol-induced CTA in adolescent male rats [22, 44–48]. These age-related differences in sensitivity to the aversive effects of ethanol are likely attributable to several contributors underlying CTA learning and/or expression rather than other physiological consequences of acute ethanol challenge such as hypothermia, sedation, or blood and brain ethanol [49]. Similarly, the results of Experiment 2 agree with previous studies that have shown attenuated CTA to ethanol following AIE exposure in adult male mice and rats [29, 30]. Importantly, attenuated sensitivity to ethanol-induced CTA is evident following different modes of adolescent ethanol exposure: intragastric [30], intraperitoneal [50], and vapor [29] exposures to ethanol during adolescence result in decreased sensitivity to the aversive effects of ethanol. While demonstrating similar insensitivity to ethanol-induced CTA, adolescents and AIE exposed adults had different patterns of neuronal activation and functional connectivity. While AIE animals weighed slightly less than water exposed animals during the adolescent exposure period (P25-P45), body weights were comparable between AIE and water-exposed rats in adulthood. Therefore, it is unlikely that the effects of AIE on adolescent body weight influenced ethanol-induced CTA in adulthood.

AIE exposed adults consumed significantly more NaCl than water exposed adults during conditioning. Possible reasons for this difference may be due to the effect of AIE on diuretic peptides such as the arginine vasopressin (AVP) system, or alterations in executive functioning neurocircuitry. Although the AVP system through AVP2 receptors regulates diuresis, limited work indicates that AVP2 expression does not differ until late adulthood [51]. Increased NaCl intake and resistance to CTA is more likely a consequence of alterations of the prefrontal cortex and executive function. Indeed, AIE exposure impairs cognitive flexibility, produces disinhibition, and increases resistance to extinction of ethanol self-administration in adulthood [52–54], with all these AIE effects linked to functional deficits in the PFC [55]. It is less likely that increased novelty seeking following AIE is a contributing factor to increased NaCl intake during conditioning and resistance to CTA, as test day intake did not differ between water- and AIE-exposed subjects injected with saline on conditioning day.

Distinct age-related differences in β-gal expression were evident in Experiment 1, with adolescents having a significantly greater number of β-gal+ cells than adults in all ROIs except for the BLA. This may reflect a greater number of excessive synaptic connections and neurons in young adolescents relative to adults prior to synaptic pruning, a hallmark of adolescent development [56]. Both ages, however, showed very limited ethanol CTA-associated changes in neuronal activation when compared with saline-injected controls across ROIs when re-exposed to the CS. In adolescents, the CeA was the only region affected by previous conditioning with the ethanol US: β-gal expression was significantly decreased in adolescents conditioned with ethanol. Given that the CeA is a hub for anxiety and alcohol circuits [57], the observed decreases in β-gal expression suggest that adolescents, but not their adult counterparts, might experience anxiolytic effects of ethanol during conditioning which resulted in decreased anxiety and attenuated activation of the CeA on test day. In contrast to adolescents, non-manipulated adults showed increases in β-gal expression in the PrL, IL, and AIV when conditioned with a 1.5 g/kg ethanol dose. This finding agrees with previously reported relationships between immediate early gene expression and aversion strength: increased cfos expression in the IC was evident in animals with moderate, but not strong CTA [58]. Furthermore, differences in cFos expression in the PrL and IL have been found following complete extinction of CTA [59], with protein synthesis inhibition in the medial prefrontal cortex (mPFC) also impairing CTA extinction [60]. Therefore, increased β-gal expression in the IC, PrL, and IL of adult males that were conditioned with the 1.5 g/kg ethanol dose may reflect, to some extent, extinction of CTA, especially given that animals were allowed to ingest NaCl for 60 minutes.

Age-related differences observed in Experiment 1 differ from those reported in the only published study that assessed cFos activation patterns associated with ethanol-induced CTA in adolescents and adults [35]. Saalfield and Spear (2019) examined cFos induction in response to re-exposure to the US (i.e., challenge dose of ethanol) following CS-US pairing and found age differences in baseline and ethanol-induced cFos expression. Specifically, under baseline conditions, adolescents had significantly less cFos positive neurons than adults in the PrL but more than adults in the bed nucleus of the stria terminalis (BNST). Adolescent-typical ethanol-induced cFos increases were evident in the NAcC and NAcSh, whereas ethanol elicited cFos expression in the BNST regardless of age. While CTA is a relatively simple task, reproducibility of altered neural activity in select brain regions across the distinct phases of the aversive conditioning process–including the US alone, CS recall following CS-US interoceptive pairing, as well as retention of neural activity during re-exposure to the US following CS-US conditioning and testing will likely pinpoint brain regions primarily involved in CTA as well as age and AIE effects therein. The Saalfield & Spear (2019) study focused on re-exposure to the US while the current study evaluated neuronal activation following exposure to the CS in the absence of a pharmacological stimulus. Indeed, exposure to the CS [61–63], US [64] or initial CS-US pairing using LiCl [65] has resulted in different patterns of neuronal activation assessed via cFos expression. In the present work, the reason for measuring β-gal (proxy of cFos) expression in response to the CS as opposed to the US (ethanol) after CS-US pairing was to avoid/reduce β-gal induction not specifically associated with the aversive effects of ethanol. Ethanol produces multifaceted effects by affecting brain circuits involved in reward, motivation, and stress [66], therefore it may be difficult to discern whether β-gal expression in response to the US is reflective of aversive or some other property of ethanol, such as level of intoxication [67, 68]. Therefore, the present study examined age differences in neuronal activation during CTA retrieval which could elicit patterns of neuronal activity different from those induced by re-exposure to the US alone. Moreover, the present study used cFos-LacZ transgenic rats on a Sprague-Dawley background as opposed to wildtype Sprague Dawley rats to investigate neuronal activation via β-gal expression. β-gal is induced only in strongly activated Fos+ neurons and not in surrounding nonactivated (Fos-) or weakly activated neurons [36].

In Experiment 2, the IL was the only brain region in which a difference in β-gal expression between AIE and water-exposed subjects occurred, with a significantly greater number of β-gal+ cells in the AIE exposed rats relative to their water exposed counterparts when collapsed across ethanol doses. It is likely that greater β-gal expression in the IL of AIE-exposed males is associated with elevated levels of anxiety in these animals, given that AIE exposure results in enhanced anxiety-like behavioral alterations [69] and the imbalance between excitation and inhibition toward excitation [70, 71], as activation of pyramidal neurons in the IL enhances anxiety responding by shifting the balance to excitation [72], Significant effects of ethanol were evident in the IL and AIV, with ethanol decreasing β-gal expression when collapsed across adolescent condition. These findings were rather unexpected, since adult non-manipulated subjects demonstrated greater β-gal expression in these ROIs following conditioning with the 1.5 g/kg ethanol dose.

Age differences (Experiment 1) and AIE effects (Experiment 2) became more apparent when the correlation analysis was used. Although this analysis revealed a circuit involving connections between the PFC (PrL, IL) and agranular insula (AI) as well as between subregions of the PFC (i.e., PrL and IL) and AI (i.e., AID and AIV) in both age groups, adolescents had substantially less functional connections between the ROIs than adults (see Fig 4a). In adults, there were two additional circuits of functional connections that were not evident in adolescent rats. The first circuit included functional connections between the IL, DS and NAc, whereas the second one included the AID, AIV and BLA. Together, these findings suggest rather limited connected involvement of cortical with subcortical brain regions in responsiveness to the CS in adolescent males relative to their adult counterparts, although cortical ROIs appear to be active regardless of age. The correlation analysis also revealed substantial differences in functional connectivity between adult AIE-exposed males and their water-exposed counterparts. As in Experiment 1, water-exposed controls showed significant positive correlations between the AIV and PFC (i.e., PrL, IL), whereas these correlations did not reach significance in AIE animals. In contrast, functional connectivity between the two subdivisions of the agranular insula (AIV and AID) was evident in AIE-exposed rats, but not in their water-exposed counterparts. Furthermore, the correlation analysis revealed a circuit involving connections between the IL, AIV, and NAcC as well as a functional connection between the IL and CeA only in water-exposed controls, with the AID and BLA functional connection evident only in AIE males. These findings suggest rather limited involvement of cortical brain regions in responsiveness to the CS following AIE.

The consistency of responsivity of IL and agranular IC in separate adult cohorts in Experiments 1 and 2 further supports their primary involvement in the interoceptive responsiveness to the CS in ethanol-induced CTA, whereas regions such as the nucleus accumbens and amygdala may have secondary involvement or are efferent targets of the primary regions. This notion is supported by established cortico-amygdalar and insular-accumbal circuits [37–41, 73–75]. Furthermore, although adolescents and AIE exposed adults demonstrated different patterns of β-gal expression and functional connectivity, the stepwise multiple regression revealed similar positive relationships between β-gal+ neurons in the AID and CS intake on test day in these animals as well as non-manipulated and water exposed adults, confirming an important role of the IC in responding to a tastant [76]. These findings are also in agreement with previous studies that have demonstrated an important role of the PFC in both the acquisition and extinction of CTA [59, 77, 78]. The results of the correlation analysis confirm previously shown involvement of the amygdala and nucleus accumbens in CTA [63]. According to Yamamoto [79], the CeA facilitates detection of the CS, whereas the BLA is implicated in the hedonic shift from positive to negative. Furthermore, synchronized activity of the PrL, agranular IC, and BLA has been shown after pairing of a tastant and LiCl [41]. In addition, functional connections between the IC and BLA regulates CTA memory allocation in the IC [37], with communication between cortico-amygdalar structures being important for CTA acquisition [38–40]. It has been shown that activation of neurons in the anterior IC projecting to the BLA in adult male mice is necessary for forming memories that associate taste with negative valence [39]. Increases of cFos have been observed in the insula, NAc (both shell and core), and BLA following pairing of saccharin and i.p. LiCl [80], suggesting functional connectivity between all these regions at least during CTA acquisition.

The main limitation of the present study is the use of only male subjects. Studies that have investigated the effect of sex on ethanol-induced CTA across age found more variability in female CTA responding. For instance, studies show more sensitivity to ethanol-induced CTA in females than males during adolescence [48], less CTA sensitivity in adulthood [24], or no difference between adult males and females [23, 61]. Our previous findings also hint at developmental exposure having no effect in females into adulthood [81]. Sex differences may also be affected by social context [15] as well as adolescent ethanol exposure [82]. Further parametric studies are needed to determine the variables that result in consistent CTA responding in females prior to evaluating age differences and AIE effects on neuronal activation in female subjects. Another limitation of the current study is that subtypes of activated neurons cannot be identified with X-gal staining. Identification of the subtypes of cells activated during re-exposure to the CS can provide valuable information about mechanisms implicated in the aversive properties of ethanol.

In summary, our results confirm relative insensitivity of adolescent male rats to the aversive effects of ethanol. This relative insensitivity of adolescents may be associated with attenuated functional connectivity between cortical regions (prelimbic, infralimbic, insular) and the amygdala and nucleus accumbens core. Exposure of male rats to ethanol during adolescence results in insensitivity to the aversive effects of ethanol later in life, with attenuated functional connectivity between the PFC, IC, and NAcC being similarly involved in this insensitivity.

## Supporting information

S1 Data (XLSX)

S2 Data (XLSX)
